# A Few Thoughts on Dental Practice

**Published:** 1854-01

**Authors:** T. W. Evans

**Affiliations:** Paris.


### SELECTED ARTICLES.

### ARTICLE XI.

*A Few Thoughts on Dental Practice.* By T. W. Evans, D. D. S., of Paris.

[The following paper, which treats on several important topics to the profession, was designed by its author, Dr. T. W. Evans, to be read before the American Society of Dental Surgeons, at their last meeting, but as he did not arrive until after the meeting adjourned, it has been furnished to us for publication.]—*Ed. Dental News Letter.*

Gentlemen—I have assumed the responsibility of addressing you upon the present occasion, not only for the pupose of contributing my mite to the general stock of information and experience, but also with a view of uniting myself more closely with an institution which has done so much for the advancement of dental science.

I here find myself surrounded by eminent men, all animated by an enthusiastic love of their profession, and attached to each other by that "unity of spirit" which is the "bond of peace;" whose names have been familiar to me almost from childhood, the memory of whom has stimulated me, both at home and abroad, to exertions which else may have been impossible. It has been my ambition to be worthy their esteem and friendship. I knew the reputation they enjoyed, not only in our own country, but in distant lands, and I resolved, when leaving home, to establish myself in what is considered to be the metropolis of medical and surgical science, to do what I could to sustain that reputation. The aim was as worthy as the task was arduous. I entered upon my mission with diffidence, but with courage, and was resolved that, so far as it was dependent upon diligent study and patient labor, it should be accomplished.

I now return to visit my native land, after six years of experience, which it would be false modesty in me to deny, have been crowned with unhoped for success. Whether that success has been deserved is not for me to decide. But this much I can certainly say, that it has not been gained by any artificial or factitious means, but has grown naturally and steadily, though rapidly, without any effort on my part except to merit it.

I have entered into no special competition with the members of our profession, more or less worthy and eminent, by whom I have been surrounded, but have always sought and succeeded in sustaining the most friendly relations with them: though I confess to have had enough of the spirit of rivalry to be resolved that neither our noble science, nor our natural reputation, should suffer at my hands.

Our reliance for success must always be upon those who have intelligence enough to appreciate, and liberality enough to reward our labors; and it is encouraging to know that, thanks to the rapid march of civilization, with which our science keeps steady pace, this class of society is ever on the increase.

Especially in this our own happy country, where the elements of not only polite learning, but of profound science are taught, even in our common schools, it may reasonably be hoped that every truly deserving profession will, ere long, be almost universally recognized.

For my own part, I have no complaint to make even in Paris, for although, as charlatanism seems to be quite as prevalent there, at the very head-quarters of science, as in less favored cities, it has offered no serious barrier to my success. Justice to my immediate "connection" requires me to say, that I have met with the most intelligent appreciation and the most liberal encouragement. The class of men who are caught by flaming advertisements, and who think a dentist has no name unless they have read it at the corners of the streets and seen it side by side with Brandreth's and Day & Martin's in newspapers, kindly pass me by on the other side. They rush to my neighbor whose liveried bill-collector struts about the city with his master's name and profession inscribed upon his back in letters of appropriate brass; or, perhaps, they patronize the "celebrated dentist" who has wheeled himself into their affection with his chariot and span of white horses!

Doubtless, gentlemen, your experience and observation have been the same. The patrons of fancy dentists, like the patrons of natural bone-setters and universal panaceas, interfere little, if any, with scientific practice. To quarrel with them is absurd. "They are joined to their idols; let them alone." As for putting down imposition, you might as well talk of putting down human nature. Mankind are used to be taken in, and they *like it.* Hudibras, who

> Know *what's what,* and that's as high
> As metaphysic wit can fly—

has taught us that

> The pleasure is as great
> In being cheated as to cheat.

Horace Mann wittily describes certain country school-houses in New England, as being very useful to the world in showing how school-houses ought *not* to be built, and it strikes me there are certain dentists in the world—in the New World as well as the Old—who do good service in showing how dentistry ought not to be practiced. It is our privilege to show the contrary; to oppose sham by science; to dispel darkness with light. And, I repeat it, it is to those who seek after the light, and who follow step by step the onward march of science, to whom we must look—and to whom, believe me, no one of us will look in vain for countenance, encouragement and support.

There may be exceptions, but I doubt, as a rule, if a diligent, pains-taking, enlightened dentist can be found, on either side the Atlantic, who can reasonably complain of want of success. He may be encompassed with difficulties; peculiar circumstances may make his path more difficult than yours—than mine; but if he has sufficient courage, and energy to oppose them, they will all be eventually removed, though to-day they lay piled across his track like mountains.

I will not dwell upon the obvious importance—the absolute necessity of labor; hard, unremitting, self-sacrificing labor in order to secure success; for on this subject we have all had line upon line, and precept upon precept; but I am sure you will pardon me if I insist that we must not only work diligently, but that we must work well.

In this country, where every man is in a hurry, where the road from the cradle to the grave is one vast race-course, and where labor is more respected and better rewarded than in any other country on the face of the globe, indolence is not so much to be guarded against as negligence.

Most men work hard enough, but few men work well enough. It is an old saying, and one that has peculiar force in our profession, that "whatever is worth doing is worth doing well." Nothing can be slighted with impunity, from the making of a pivot to the delicate adjustment of a spring. Whether our workmanship is to be exposed to the eye, or concealed from sight, it ought to be done with an artistic nicety of finish. The filling of a tooth, for example, once thought by many to be so simple and trifling a matter, requires as much care as the setting of precious stones.

I mention this only as one instance, but it is one peculiarly significant to me, because it was in performing this operation that I first became aware of the importance of doing the operative, or rather the artificial part of our work, with the utmost fidelity. I had the good fortune to learn this lesson at the outset of my career, and the first testimonial I received in my profession, though much less brilliant and costly than many I have since received, I value more than all the rest—more even than those coming from royal hands—because it was a public recognition from my own countrymen, from my own colleagues even, not of the many things which I had done indifferently, but of the one thing I was supposed to have done well, and that one thing, gentlemen, was the filling of teeth, and I cannot tell you with what pride I have since learned that, in consequence of this tribute to acknowledged merit, some of my young contemporaries have been stimulated to apply the same degree of labor and care to other branches which I had devoted to this one branch of our art.

I have thought it not amiss, gentlemen, in these fast, labor-saving times, to dwell with some emphasis on the essential importance of executing the minutest details of our work with the greatest care and skill; and if I have ventured to illustrate my idea by a passing allusion to my own experience, it is not for the purpose of making an invidious comparison between myself and others; but, on the contrary, to show if I had met with any peculiar success it has resulted not from any talent peculiar to myself, but from a capacity common to us all: viz. the *capacity of hard and faithful labor.*

In addition to the more obvious reasons for neglecting no detail of our workmanship, I may mention that by rigidly following this course, (I speak again from my own experience,) the humblest of us may hope to arrive at some discovery or improvement for the advancement of the profession.

Dr. J. H. Foster, in his excellent opening address before this society, at its annual meeting in 1851, remarked: "Those who are attaining pre-eminent rank and reputation in our profession, are those who are performing new and untried operations."

Now, who of us does not know that these "new and untried operations" are often suggested by the faithful performance of those which are old and familiar.

The new idea comes to us, as it were, like a spark from an anvil in the heat of our work, and seems to endow us for the moment with a new sense. We work on with renewed zeal; the vague idea gradually assumes form and distinctness; until at last we are enabled to present our science with a new discovery, baptized with the sweat of our brow, if not christened by the world with our name.

I have been experimenting in my humble way, and "performing new and untried operations" from the beginning; knowing well that *where nothing is* ATTEMPTED *nothing is* ATTAINED.

When I was told, years ago, (to recur to my favorite experience,) that there were certain teeth so decayed, and their cavities so large, that to fill, and thus to save them, was next to impossible, I was all the more determined to persevere; and after applying myself diligently to the work for many long and weary months, my efforts were at last crowned with success, and rewarded with approval. Animated by this early and triumphant achievement, (for I confess to have been proud of it,) I was ambitious to excel in other departments of our art, and was even presumptuous enough to covet a wider and more difficult sphere for my labors.

Indeed, it had been the dream of my youth to go abroad; so, gathering up the few laurels I had earned—scarcely enough to make a chaplet—and calling into exercise all my courage, I crossed the ocean, and established myself boldly in the heart of the Old World.

There, as here, my determination was an entire devotion of myself to my calling. I own to having had at first some little misgivings—as any one might well have on finding himself for the first time amidst all the splendor and glory of the most brilliant capital in Europe—but yet something within me—it was probably a little of the old American leaven—made me feel that I should succeed. This, however, is not very striking, for somehow or other there appears to be something in the heart of every American which tells him pretty much the same thing.

But I am indulging too much in personal reminiscences. To go back a little: Believing with Dr. Foster in the importance of "new and untried operations," and feeling that our own science, like every other, was yet to be enriched by many discoveries, I was anxious to have my share in their discovery and application.

It was at this period, gentlemen, that some of the most respectable members of our profession were seeking to find out some method whereby, in certain exceptional cases, teeth might be filled with less labor for the operator and less annoyance to the patient than the ordinary method of filling with gold.

I was not without hope, myself, that some such discovery would be made; not that I was at all desirous to shrink from any necessary amount of labor; but I *was* anxious that a large class of persons, whose nervous constitutions can poorly stand the fatigue of a prolonged operation, should, so far as was safe and practicable, be spared the torment of undergoing them. I saw at once, it was true, that most of our fillings must continue to be done in the old way; but I had hoped that certain peculiar cases might be treated with equal success in some less fatiguing and more expeditious manner. And, at one moment, as some of you, gentlemen, are aware, I fancied that I myself had done something towards the proposed discovery; and, over anxious, perhaps, to extend its benefits, I was too easily induced to proclaim it to the world, and associate it with my name. I did not do this without numerous and apparently successful experiments; and the experience of other and eminent men seemed to show that I had conferred upon our science an important benefit. But the final result proved me to have been in error; and I had no honorable recourse but to publish *this fact* as widely, as a short time previous I had published my supposed discovery. I promptly and frankly adopted this course, and have never *repented it.—Dent. News Letter.*

[To be continued.]

